# The distinct functions of MIF in inflammatory cardiomyopathy

**DOI:** 10.3389/fimmu.2025.1544484

**Published:** 2025-02-28

**Authors:** Kyle R. Pressley, Yashfa Naseem, Saisha Nalawade, Thomas G. Forsthuber

**Affiliations:** ^1^ Department of Molecular Microbiology and Immunology, University of Texas at San Antonio, San Antonio, TX, United States; ^2^ Department of Neuroscience, Developmental and Regenerative Biology, University of Texas at San Antonio, San Antonio, TX, United States; ^3^ Department of Pre-clinical Immunology, Corner Therapeutics, Watertown, MA, United States

**Keywords:** MIF, inflammatory cardiomyopathy, dilated cardiomyopathy, viral myocarditis, autoimmune myocarditis, cardiac inflammation

## Abstract

The immune system plays a crucial role in cardiac homeostasis and disease, and the innate and adaptive immune systems can be beneficial or detrimental in cardiac injury. The pleiotropic proinflammatory cytokine macrophage migration inhibitory factor (MIF) is involved in the pathogenesis of many human disease conditions, including heart diseases and inflammatory cardiomyopathies. Inflammatory cardiomyopathies are frequently observed after microbial infection but can also be caused by systemic immune-mediated diseases, drugs, and toxic substances. Immune cells and MIF are implicated in many of these conditions and may affect progression of inflammatory cardiomyopathy (ICM) to myocardial remodeling and dilated cardiomyopathy (DCM). The potential for targeting MIF therapeutically in patients with inflammatory diseases is an active area of investigation. Here we review the current literature supporting the role(s) of MIF in ICM and cardiac dysfunction. We posit that future research to further elucidate the underlying functions of MIF in cardiac pathologies is warranted.

## 1 Introduction

The immune system plays a critical role in cardiac homeostasis and disease, and the innate and adaptive immune systems can be beneficial or detrimental in cardiac injury (1, 2). Inflammatory cardiomyopathies represent a broad group of disorders with heterogenous etiology characterized by cardiac dysfunction primarily caused by myocardial inflammation and ventricular remodeling (2, 3). ICM is frequently observed after viral infections, but can also be caused by bacterial, protozoal, or fungal infections as well as a wide variety of systemic immune-mediated diseases, autoimmune disease directly affecting the heart (e.g., autoimmune myocarditis), drugs, and toxic substances (2, 3). Myocardial remodeling can occur within the spectrum of inflammatory cardiomyopathies, as well as heart disease associated with secondary immune responses (e.g., myocardial ischemia), and lead to dilated cardiomyopathy, increased diastolic pressure due to reduced ejection fraction, myocardial hypertrophy, and fibrosis (1). Extensive research efforts have improved diagnosis and understanding of ICM pathogenesis, but when complicated by arrythmia, left ventricular dysfunction, or heart failure (HF), it is still associated with a poor prognosis (3–5).

The pleiotropic proinflammatory cytokine MIF is implicated in several cardiac pathologies, including ICM and HF (6). Evidence suggests that MIF has diverse functions in cardiac disease, largely depending on the underlying etiology. In the following, we will review current knowledge regarding the functions of MIF in ICM, with additional focus on cardiac pathologies that share overlapping features with ICM.

## 2 MIF

MIF is a pleiotropic proinflammatory cytokine involved in various cellular and immune functions, including leukocyte recruitment, immune responses, counter-regulation of glucocorticoids (GCs), cell proliferation, and tumorigenesis (6). It is a highly conserved homotrimer of ~12.5 kDa that first gained attention for its role in tissue macrophage retention, and later as an *in vitro* surrogate for delayed-type hypersensitivity (7, 8). Shortly after the structure of MIF was determined, a second MIF family member was identified, D-DT (MIF-2) (9, 10).

Initially identified as a T cell-specific cytokine, MIF was later determined to be secreted by several distinct cell types (6, 11). The role of MIF in the immune response generated ongoing efforts to further elucidate its multifaceted functions in human health and disease.

### 2.1 MIF signaling and function

MIF signals in an autocrine and paracrine manner via binding to cognate (i.e., CD74/CD44) and non-cognate (i.e., CXCR2, CXCR4, and CXCR7) receptors on the cell surface, as well as via direct interactions with intracellular proteins (12). Cognate MIF signaling occurs through a receptor complex composed of the ligand-binding protein CD74 and the signal transducer CD44 (11, 12). Upon binding of MIF, horizontal membrane recruitment of CD44 is initiated by CD74, followed by phosphorylation of their cytosolic domains initiating downstream signal transduction (13). Downstream signaling pathways, including ERK1/2 and PI3K-AKT pathways, are triggered via phosphorylation of SRC by PKA (12, 14). AMPK is also phosphorylated, but the exact signaling pathway is unknown (12, 15). Additionally, CD74 can be cleaved in a CD44-dependent manner by the SPPL2A protease to generate a CD74 intracytoplasmic domain (11). In B cells, this process results in activation of PI3K-AKT, NF-κb, and anti-apoptotic signaling pathways (11). These cognate receptor-mediated signaling pathways promote cell proliferation, migration, and survival, as well as increased glucose uptake and fatty acid oxidation (12).

In non-cognate MIF receptor signaling, CD74 associates with CXCR2, CXCR4, and/or CXCR7 (12, 16). MIF signal transduction through CXCR2 or CXCR4 requires interaction with CD74 and triggers a classical G-coupled protein pathway, resulting in stimulation of ERK1/2, PI3K-AKT, and PLC-β signaling cascades (12). These signaling pathways promote cell proliferation, migration, and survival, as well as increased cytosolic Ca^2+^ (12). Importantly, due to internalization of CXCR2 and CXCR4, macrophage retention is promoted within inflamed tissues (16). Moreover, MIF is a ligand for CXCR7, for which two downstream G-coupled protein pathways have been described (12). CXCR7-associated MIF signaling is negatively regulated by β-arrestin, and promotes cell proliferation, migration, and survival (12). Importantly, MIF-2 also activates CD74 but lacks the pseudo-(E)LR motif present in MIF (17, 18). Based on computational modeling accounting for this absent motif, MIF-2 does not engage CXCR2, CXCR4, or CXCR7 (12, 17, 18). Interestingly, MIF was reported to bind and inhibit epidermal growth factor receptor (EGFR), blocking the activation of EGFR-induced ERK1/2 and c-Jun signaling (19).

Intracellularly, MIF binds and inhibits JAB1/CSN5 and p53, promoting cell cycle progression and preventing apoptosis (11, 12). Upon binding of MIF to CD74, β-arrestin interacts with CD74 resulting in CD74-mediated endocytosis of MIF and sustained ERK activation (20). Additionally, MIF is required for the interaction between vimentin and NLRP3, which is essential for NLRP3 activation and for the rate-limiting proteolytic cleavage of IL-1 family member precursors to produce active cytokines (i.e., IL-1β and IL-18) (21, 22). Activation and differentiation of innate and adaptive immune cells into distinct subsets is promoted by these interleukins, and thus, MIF can indirectly regulate this process. Moreover, MIF can function as an endonuclease by interacting with apoptosis-inducing factor and translocating to the nucleus, where MIF cleaves genomic DNA and promotes cell death (23). Additional intracellular binding partners of MIF include superoxide dismutase, Jun-c activation domain-binding protein, hepatopoietin, ribosomal protein S19, Gremlin-1, High temperature requirement A1, Thioredoxin-1, thioredoxin-interacting protein, and insulin (24). Furthermore, MIF functions as a phenyl pyruvate tautomerase and catalyzes the conversion of the D-isomer of D-dopachrome to DHICA (25). However, the precursor of D-dopachrome, D-Tyrosine, is not synthesized in vertebrates. Post-translational modifications can render MIF enzymatically inactive without affecting its immunomodulatory functions (26). Therefore, the biological role of this tautomerase activity in vertebrates is unclear (12).

MIF plays a critical role in mediating resistance to pathogens and promoting several types of immune and autoimmune diseases (12). In response to various stimuli, MIF is secreted via an ABC transporter-dependent export pathway (27). MIF promotes the production of pro-inflammatory cytokines (e.g., TNF-α), reactive oxygen species (ROS), and influx of leukocytes into inflamed tissues (11, 28, 29). Additionally, MIF signaling upregulates Toll-like receptor 4 (TLR4) and dectin-1 to regulate the innate immune response (30, 31).

A unique property of MIF is that it can counteract glucocorticoid-induced suppression of proinflammatory cytokines and this effect is mediated by acting on several important regulatory steps in the inflammatory response (11). MIF binds the NF-κb inhibitor (IκB) and MAPK phosphatase-1 (MPK1), overriding glucocorticoid-induced expression of these proteins (11). Importantly, secretion of MIF is induced by glucocorticoids in a tightly regulated manner; low physiological concentrations of glucocorticoids stimulate MIF release from monocytes, macrophages and T cells, whereas high, anti-inflammatory concentrations of glucocorticoids prevent MIF secretion (32–34). Accordingly, inhibition of MIF has been proposed as a potentially effective therapeutic strategy for the treatment of inflammatory and autoimmune diseases, in particular in conditions associated with glucocorticoid resistance or glucocorticoid dependence (11).

A growing body of evidence supports the beneficial role of MIF in regulating the immune response and microbial clearance, renal protection, maintenance of immune-privileged sites, wound healing, insulin secretion, and in some cases protection of cardiac and nervous system tissues (6). However, as will be discussed in the next section, MIF is also involved in the pathogenesis of disease, influences disease severity, and is associated with several inflammatory and autoimmune diseases.

### 2.2 MIF in human disease

MIF plays a critical role in many human disease conditions, including sepsis, viral infections, diabetes, and cancer (35). In sepsis, it has been reported that patients with significantly higher levels of MIF in plasma exhibited an increase in fatal outcomes (36). In an experimental model of sepsis, neutralization of MIF by anti-MIF antibodies and genetic ablation of MIF afforded protection (37). Moreover, administration of recombinant MIF significantly enhanced LPS-induced lethality in an experimental model of sepsis (38), further supporting the role of MIF in the pathogenesis of septic shock. MIF is also associated with viral infections such as HIV, Influenza, Dengue, Ebola, cytomegalovirus, Japanese encephalitis, and West Nile virus (6). In Type 2 diabetes (T2D), patients’ levels of circulating MIF increase, and the higher blood concentration of MIF has been associated with an increased risk of developing T2D (39, 40). Several cancers are characterized by overexpression of MIF which contributes to immune evasion, tumor growth and angiogenesis, and metastasis (6, 41–43).

Initial studies examining the role of MIF in autoimmune disease were largely focused on studying its expression in rheumatoid arthritis (RA) patients (44). Increased levels of MIF were reported in the circulation, synovial fluid, and inflamed synovial tissue in RA patients, providing evidence of the proinflammatory function and regulation of glucocorticoids by MIF (44, 45). Neutralization or genetic deletion of MIF in experimental animal models of RA resulted in reduced expression of proinflammatory cytokines and reduced disease, providing evidence for the contributory role for MIF in RA pathogenesis (46–49). Furthermore, evidence for the role of MIF in the development, pathogenesis, and treatment of SLE, spondyloarthritis, juvenile idiopathic arthritis, granulomatosis with polyangiitis, Type 1 diabetes, multiple sclerosis, and autoimmune myocarditis have been reported (6, 11, 50–52).

Taken together, MIF plays a significant role in the pathology of several diseases, further supporting the ongoing effort to develop therapeutic strategies targeting MIF.

## 3 Inflammatory cardiomyopathy

### 3.1 Pathogenesis of inflammatory cardiomyopathy

ICM is characterized by inflammatory cell infiltration in the myocardium, has a varied etiology, and carries a high risk of deteriorating cardiac function (2, 3). Inflammatory cardiomyopathies are comprised of a heterogenous group of conditions that include infectious, autoimmune (i.e., autoimmune myocarditis), and inflammatory heart conditions.

The pathogenesis of ICM is complex, involving both innate and adaptive immune responses. Immune cells such as T cells, B cells, monocytes, dendritic cells (DCs), and natural killer (NK) cells infiltrate the myocardium and mediate cardiac tissue damage (3). ICM is frequently observed after viral infections, for example coxsackievirus B virus, Epstein-Barr virus, or Covid-19 virus infection, but can also result from autoimmune diseases such as connective tissue disease and systemic lupus erythematosus (SLE), as well as from exposure to a wide variety of toxic substances and drugs (2, 3). Regardless of the specific etiology of ICM, infiltrating immune cells secreting proinflammatory cytokines, including IL-17, TNF-α, IL-6, and IL-1β, perpetuate inflammation and tissue damage (3, 53).

Genetic and environmental factors are frequently implicated in the development and progression of ICM. A genome-wide association study revealed a risk locus for DCM encoding certain HLA Class I and HLA Class II alleles, suggesting a role for genetically driven, and possibly autoimmune inflammatory processes in the pathogenesis of idiopathic DCM (54). Cardiac myosin-specific Th17 cells imprinted in the intestine by *Bacteroides* were required for progression to fatal heart disease in a mouse model of spontaneous autoimmune myocarditis (55). Moreover, patients with ICM have detectable immune reactivity to both myosin 6 antigens and myosin peptide mimics derived from commensal *Bacteroides* species from the gut (55). Thus, evidence suggests that the gut microbiome can be important for restraining pathogenic T cells in autoimmune myocarditis.

The complications of ICM include DCM, increased diastolic pressure and reduced ejection fraction, myocardial hypertrophy, and fibrosis as strong risk factors for HF (1, 4, 5), highlighting the importance of effective therapeutic interventions for ICM.

### 3.2 MIF signaling in cell types implicated in inflammatory cardiomyopathy

**Figure 1:**
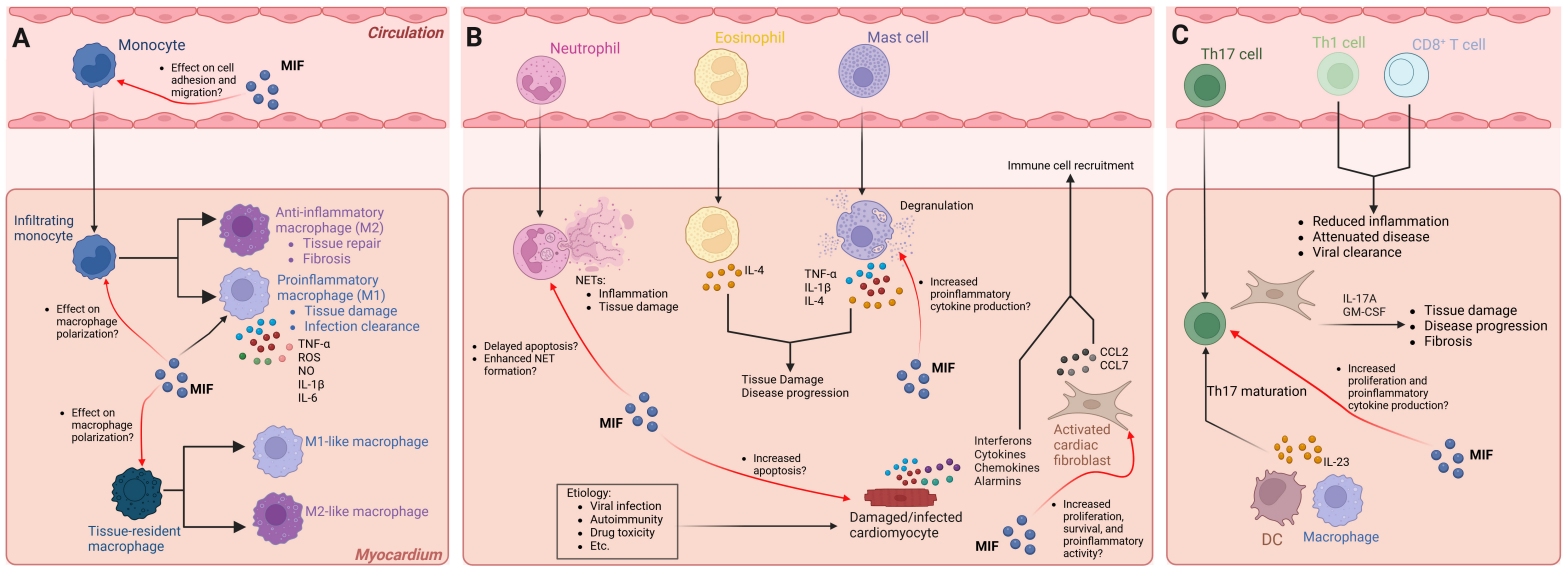
Cellular responses of infiltrating immune cells and the effect of MIF signaling in ICM pathogenesis. Several innate and adaptive immune cells, as well as resident cardiac cells, contribute to ICM pathogenesis and progression. MIF signaling affects different cell types under physiological and pathological conditions. The exact role(s) of distinct cell types may be etiology specific. The mechanisms illustrated reflect all forms of ICM. **(A)** Monocyte subsets infiltrate the myocardium and polarize into proinflammatory (M1) or anti-inflammatory (M2) macrophages. MIF promotes proinflammatory macrophage function, influences macrophage polarization, and promotes adhesion to endothelial cells; however, whether MIF influences macrophage polarization and migration of infiltrating monocytes and/or tissue resident macrophages in ICM has yet to be elucidated. **(B)** Neutrophils, eosinophils, and mast cells infiltrate the myocardium and promote disease onset and/or progression. MIF signaling in neutrophils delays apoptosis and promotes NET formation, potentially contributing to ICM severity. MIF signaling in mast cells promotes fibrosis; however, whether MIF signaling in mast cells promotes the development of ICM and progression to cardiac fibrosis is unclear. Damaged or infected cardiomyocytes undergo apoptosis, and in the process, release signaling molecules that promote immune cell infiltration. MIF was shown to induce cardiomyocyte apoptosis, but whether this occurs in ICM is unknown. Activated fibroblasts promote immune cell infiltration. MIF signaling in cardiac fibroblasts promotes survival, proliferation, and proinflammatory activity, but whether this contributes to immune cell infiltration in ICM remains unclear. **(C)** Pathogenic Th17 cells infiltrate the myocardium and promote disease progression in part by IL-23 secreted from antigen presenting cells, whereas Th1 and CD8+ T cells attenuate ICM. MIF is essential for T cell proliferation and cytokine production, but whether MIF signaling in Th17 cells promotes disease progression has yet to be elucidated. Black arrows indicate disease processes validated in experimental models and human studies. Red arrows indicate a potential role for MIF signaling that has not been directly tested. NET, neutrophil extracellular trap; DC, dendritic cell. Graphics created with BioRender.com.

In ICM, cell types of the innate and adaptive immune system play critical roles in mediating inflammation and tissue damage. Much of our understanding of how immune cells contribute to inflammatory cardiomyopathies came from studies in experimental animal models (1). These studies revealed that cells of the innate and adaptive immune system, as well as resident cardiac cells including fibroblasts, endothelial cells, and cardiomyocytes play a significant role in the pathogenesis of ICM (1, 3). Robust evidence supports a potential role for MIF signaling in ICM. Accordingly, there is an ongoing effort to determine how MIF signaling in these cell types influences ICM pathogenesis (**Figure 1**).

#### 3.2.1 Innate immune cells

In virus-induced ICM, activated innate immune cells and cardiac cells release proinflammatory mediators, resulting in further activation and migration of additional innate immune cells to cardiac tissue, including monocytes, macrophages, mast cells, neutrophils, and DCs (3, 56). The main inflammatory cell subsets found in human and experimental myocarditis are monocytes and macrophages (3, 56). In monocytes, MIF upregulates the adhesion molecules VCAM-1 and ICAM-1 enhancing cell adhesion to endothelial cells (14), and inhibits migration through the CXCR4 receptor, independent of CD74, by modulating Rho GTPase activities (57). Further, MIF sustains proinflammatory functions in macrophages by inhibiting p53-dependent apoptosis and stimulating production of TNF-α, nitric oxide, and reactive oxygen species (ROS) (58–60). MIF modulates toll-like receptor 4 expression (61), and induces matrix metalloproteinase-9 expression (62). Still, how MIF signaling in monocytes and macrophages influences ICM pathogenesis remains unclear (**Figure 1A**). Of note, monocytes recruited to the myocardium in response to cardiac injury can differentiate into macrophages that polarize into either proinflammatory (M1) or anti-inflammatory (M2) phenotypes depending on the signals they receive. M1 macrophages contribute to tissue damage and T cell activation through the secretion of inflammatory cytokines such as TNF-α, IL-1β, and IL-6, while M2 macrophages are involved in tissue repair and fibrosis (63–66). MIF has been demonstrated to influence macrophage polarization (67, 68), suggesting its potential role in ICM. Moreover, cardiac tissue injury promotes the polarization of CCR2-negative cardiac tissue resident macrophages into M1-like or M2-like macrophages (66), but how MIF influences this process remains unclear (**Figure 1A**). Considering that MIF can promote both M1- and M2-associated macrophage functions, several factors including monocyte subtype, tissue specificity, signaling molecules, and presence of infectious microbes likely influence the effect of MIF on macrophage polarization.

Additional innate immune cells contributing to ICM pathogenesis include neutrophils, mast cells, eosinophils, NK cells, and DCs. Neutrophils are the first responders within the group of infiltrating innate immune cells and their proportion among cardiac inflammatory cells is strongly associated with the severity of ICM (3). Studies in mice with experimental autoimmune myocarditis (EAM) revealed that neutrophils can sustain inflammation via neutrophil extracellular traps (NETs), and the severity of myocarditis in these mice is strongly associated with neutrophil accumulation in cardiac tissue (69, 70). MIF delays neutrophil apoptosis (71) and enhances neutrophil antimicrobial activity by promoting NET formation (72). Thus, current evidence suggests a potential role for MIF in promoting ICM by influencing neutrophil function (**Figure 1B**).

Mast cells are among the first cells to respond to viral infections of cardiac tissue and the degranulation process results in the production of proinflammatory cytokines including TNF-α, IL-1β, and IL-4 (3). Interestingly, cardiomyopathy can be a complication in patients with eosinophilia, and studies in mice with EAM implicate eosinophil-derived IL-4 in the progression of ICM to DCM (73). MIF can promote mast cell recruitment (74), and in fibrotic diseases, mast cell-derived MIF stimulates fibroblast proliferation and collagen production (75). Still, whether MIF signaling in mast cells influences the development of ICM and progression to cardiac fibrosis has yet to be established (**Figure 1B**).

NK cells and DCs are also recruited to the heart in response to infection. It has been suggested that DCs processing and presenting endogenous antigens liberated during infections might trigger autoimmune myocarditis, possibly via determinant (epitope) spreading (3, 76). However, DCs and NK cells may also have a role in preventing the development of ICM and progression to HF (77–79). In NK cells, MIF can reduce cytolytic activity and prevent the release of perforin granules (80, 81). In DCs, MIF promotes migration (82), activation and proinflammatory cytokine production (83), maturation of CD11b^+^ DCs, and differentiation of Ly6C^hi^ monocytes into TNF- and iNOS-producing DCs (84). Thus, MIF signaling has several distinct functions in NK cells and DCs, but how these processes influence ICM pathogenesis has yet to be elucidated.

#### 3.2.2 Adaptive immune cells

T cells and B cells play critical roles in cardiac homeostasis and repair and are key mediators of cardiac damage in experimental animal models of myocarditis (85–89). Activated T cells are thought to be major contributors to the pathophysiology of autoimmune ICM (90). Further, in mice with coxsackievirus B3 (CVB3)-induced myocarditis, CD4^+^ T cells are critical for disease (85), whereas CD8^+^ T cells can attenuate disease (91). Deletion of T-bet, a transcription factor required for Th1 lineage differentiation and IFN-γ production, increased the severity of EAM by inducing IL-17 production (92). Furthermore, Th17 cells promote progression of ICM to DCM through their effects on cardiac fibroblasts (86) (**Figure 1C**). This is in part facilitated by IL-23 secreted from antigen presenting cells that promotes the maturation of Th17 cells (93). Indeed, IL-23 has been shown to be essential for the initiation of GM-CSF-driven autoimmune myocarditis (94, 95). MIF is essential for T cell proliferation and cytokine production (96, 97), but the exact mechanism by which MIF signaling in T cells influences ICM remains unclear (**Figure 1C**). Regulatory T cells (Tregs) protect against myocarditis (64), and MIF deficient mice show impaired immunosuppressive function with lower expression of IL-10 and TGF-β (98). Furthermore, CD69 regulates the Foxp3-ROR-γt pathway to promote Treg differentiation (89). In models of myocarditis and DCM, CD69-deficient hearts show altered Treg-Th17 immune cell infiltration and Foxp3/ROR-γt signaling, further supporting a role for imbalanced Treg-Th17 ratios that contribute to ICM pathogenesis (89). Thus, the current evidence suggests that MIF is essential for T cell activation and regulation and implicates MIF signaling in diseases associated with a dysregulated immune response, including ICM.

The role of B cells in ICM is less clear. Accordingly, most of the information regarding B cells in ICM comes from the observation of autoantibodies in DCM patients (3, 99, 100). Additionally, depletion of CD20^+^ B cells has shown efficacy in patients with ICM who did not respond to steroid-based therapy (101), suggesting a pathogenic role for B cells. Furthermore, MIF influences B cell chemotaxis and survival (102, 103), suggesting that MIF may promote pathogenic B cell functions in ICM. Nevertheless, future research is needed to better understand how MIF signaling can influence B cell functions in ICM.

#### 3.2.3 Resident cardiac cells

Resident cardiac cells including fibroblasts, endothelial cells, and cardiomyocytes play a significant role in ICM pathogenesis (1, 3). In mice with myocarditis, cardiac fibroblasts secrete cytokines that promote the migration of monocyte subsets to the myocardium and facilitate the differentiation of these monocyte subsets into macrophages (63, 104). MIF promotes cardiac fibroblast proliferation through the Src kinase signaling pathway (105), induces proinflammatory gene expression during myocardial infarction (MI) (106), and promotes survival via CXCR4/AKT signaling (107). These findings suggest a complex role for MIF signaling in cardiac fibroblasts and highlight the need for future research (**Figure 1B**).

Upon infection, cardiomyocytes are activated and release cytokines, chemokines, interferons and alarmins, resulting in the activation and migration of innate immune cells to the myocardium (3). Infection of endothelial cells with parvovirus B19 triggers the release of proinflammatory cytokines thereby inducing cardiomyocyte apoptosis (108). MIF has been demonstrated to induce cardiomyocyte apoptosis (109); however, it has also been shown to promote glucose uptake and reduce the expression of pro-fibrotic genes under ischemic conditions (106, 110). Interestingly, evidence suggests that MIF stimulation does not induce proinflammatory gene expression in cardiomyocytes. Nevertheless, future research should aim to elucidate how MIF signaling in cardiomyocytes influences ICM developing from different etiologies.

## 4 MIF in inflammatory cardiomyopathy, cardiac remodeling, and heart failure

Evidence suggests that MIF has diverse functions in ICM, cardiac remodeling, and HF. The diverse functions may be attributable to etiology. The evidence discussed in this section relating to cardiomyopathy and HF is summarized in **Table 1**.

**Table 1:** Effect of MIF signaling in inflammatory cardiomyopathy, cardiac remodeling, and heart failure.

Condition	Effect of MIF	Model/Data Type
Inflammatory Cardiomyopathy		
Chagas disease	↓	• BALB/c mice infected with *T. cruzi* (111, 112) • *In vitro* (macrophages infected with *T. cruzi*) (60)
CCC	↑	• Clinical correlation (59) • C3H mice infected with *T. cruzi* (59)
Viral myocarditis	↑	• BALB/c mice infected with CVB3 (113)
Sepsis-induced cardiomyopathy	↑	• Wister rats injected with LPS (114) • CD74^-/-^ and WT mice injected with LPS (115)
Iatrogenic myocarditis	↓	• C57BL/6 MIF^-/-^ and WT mice injected with doxorubicin (116)
Toxin-induced myocarditis	↑	• C57BL/6 CD74^-/-^ and WT mice injected with ethanol for three consecutive days (117)
Autoimmune myocarditis	↑	• Lewis rats with EAM (50, 118)
Heart Failure and Cardiac Remodeling		
Heart failure	↑	• Clinical correlation with fibrosis (119) • Clinical correlation with pulmonary hypertension and death (120, 121)
	↓	• *In vitro* (cardiac myofibroblasts treated with sCD74 and MIF) (107)
Pressure-overload induced cardiac hypertrophy	↓	• C57BL/6 MIF^-/-^ and WT mice subjected to transverse aortic coarctation (122) • MIF^-/-^ and WT mice subjected to abdominal aorta constriction (123)

WT, wildtype; EAM, experimental autoimmune myocarditis; CVB3, Coxsackie virus B3; LPS, lipopolysaccharide; sCD74, soluble CD74; CCC, chronic chagasic cardiomyopathy. ↓, protective; ↑, detrimental.

### 4.1 Infectious inflammatory cardiomyopathy

Epidemiologic and experimental evidence suggests an important role for MIF in infectious and para-infectious heart conditions, including Chagas disease, rheumatic heart disease (RHD), sepsis, and viral-mediated heart diseases.

Chagas disease (*American trypanosomiasis*) is a leading cause of cardiomyopathy in Latin America, with approximately 30% of patients exhibiting cardiomyopathy (124). Chagas disease is caused by the protozoan parasite *Trypanosoma Cruzi*. Chronic chagasic cardiomyopathy (CCC) is the most common worldwide cause of infectious cardiac pathology (125). It affects approximately 30% of the infected individuals and is characterized by heart inflammation and dysfunction. *T. cruzi* invades and multiplies within macrophages and cardiac myocytes (126). The host immune response leading to the recruitment and migration of inflammatory cells to the myocardium during infection involve cytokines, chemokines and adhesion molecules (127).

Knockout mice lacking proinflammatory cytokines such as IL-12, TNF-α and IFN-γ are unable to control parasitemia, indicating their crucial role in protective immunity to *T. cruzi* (60, 128). IL-12 enhances IFN-γ secretion by NK cells which induce nitric oxide (NO)-dependent macrophage microbicidal activity of *T. cruzi* leading to a protective Th1 response (129–131).

Notably, MIF is involved in host resistance to several parasitic infections and acts upstream of several proinflammatory cytokines (132). MIF induces early dendritic cell maturation and IL-12 production by potentially activating the p38 pathway (132). Early MIF induction by macrophages in chagas disease has been reported to play an important role in providing resistance against *T. cruzi* infection by promoting the production of IL-12, IL-18, IL-6, TNF-α, IL-1β, IFN-γ, NO and ROS during acute infection (111, 112).

However, additionally MIF may be involved in the pathophysiology of CCC. Impaired cardiac function correlates with increased circulating levels of MIF in patients with CCC (59). Chronically infected mice display progressive MIF overexpression in cardiac myocytes, leading to intense inflammatory infiltration which contributes to parasite-triggered ICM (59). CD8^+^ T cells are the predominant inflammatory cell population known to infiltrate the myocardium during CCC (59, 133, 134).

Exacerbated inflammation is involved in cardiomyocyte destruction leading to fibrosis of the myocardial tissues and progression to DCM (135, 136). Additionally, TNF-α and ROS play pathogenic roles in CCC as demonstrated by data from parasite-infected murine macrophage cultures treated with MIF (59). Mechanistically, the MIF-CD74 complex is internalized through endocytosis resulting in activation of NADPH oxidase and generation of ROS (28, 60). Several reports suggest that chagasic myocardia are exposed to sustained oxidative stress-dependent injuries that might contribute to pathogenesis of CCC (137, 138). The major clinical manifestations of CCC include heart failure, cardiac arrhythmias, heart block, thromboembolism, chest pain syndrome and sudden death (139, 140).

Anti-trypanosomal treatment is recommended for all patients with acute, congenital, and reactivated infections in order to eradicate the parasite. But this treatment is not indicated in patients with advanced heart failure from Chagas disease (139). Implantable cardioverter-defibrillators are considered to prevent sudden cardiac death in high-risk patients, and heart transplantation is required for patients with refractory end-stage heart failure (141). Importantly, assessing circulating MIF levels could provide an additional tool for identifying chronic chagasic patients. The analysis of different cytokines present in the cardiac tissue and in the bloodstream during *T. cruzi* infection and their correlation with the degree of myocardial damage may be beneficial in developing novel therapies aimed to control morbidity and mortality in chagasic patients (59).

Rheumatic fever represents another key mechanism in the context of immune-mediated cardiac damage. It is thought to arise as an autoimmune response triggered by an infection with group A Streptococcus (GAS), wherein immune cross reactivity (molecular mimicry) between GAS antigens and cardiac tissue results in inflammation, including that of the heart valves (142). This may result in chronic RHD characterized by myocarditis and damage to heart valves and associated complications (142). Although no functional studies are available, a study in Saudi Arabian patients reported that non-carriers of the 173C MIF allele have an increased risk of RHD, whereas presence of this allele was associated with late disease onset (143). The same study reported the 794 6-repeat allele to be associated with increased risk of RHD, whereas the 794-5 repeat allele was associated with a decreased risk (143). This study suggests that MIF plays a pathogenic role in RHD, but future studies are needed to elucidate the underlying molecular mechanisms.

Viral myocarditis induced by CVB3 can also result in ICM (144). Notably, serum MIF concentrations were significantly increased 7-14 days post-infection in a mouse model of viral myocarditis after CVB3 infection (113). Treatment of these mice with an anti-MIF antibody increased survival, decreased disease severity, and decreased IL-1β and TNF-α in the myocardium (113), suggesting MIF inhibition as a viable treatment option for dampening the inflammatory response in viral myocarditis.

Cardiomyopathy is a common complication of sepsis (145). In a rat model of sepsis, treatment with neutralizing anti-MIF reduced acute inflammation of the heart and improved myocardial dysfunction, providing evidence that MIF inhibition may be beneficial in sepsis-induced cardiomyopathy (114). Using the same model in mice, CD74 ablation protected against sepsis-induced cardiomyopathy (115), providing further evidence that MIF promotes cardiomyopathy in sepsis.

Currently, the data suggests that MIF plays a harmful role in cardiomyopathies caused by CV3B infection, RHD, Chagas disease, and sepsis, but how MIF signaling affects the pathogenesis of inflammatory cardiomyopathies caused by other infectious and para-infectious heart diseases has yet to be elucidated.

### 4.2 Iatrogenic and toxin-induced inflammatory cardiomyopathy

ICM can be iatrogenic or toxin-induced (2). In a model of doxorubicin-induced cardiomyopathy, MIF deficiency exacerbated cardiomyopathy and mortality, suggesting its cardioprotective role (116). Alcoholism carries a high incidence of cardiac morbidity and mortality due to the development of alcoholic cardiomyopathy (146). Alcoholic cardiomyopathy is not classified as ICM; however, it shares many characteristics regarding cardiac dysfunction. Interestingly, CD74 ablation counteracts alcohol-induced myocardial dysfunction by ameliorating inflammation and apoptosis, possibly through autophagy mediated by AMPK signaling (117). Thus, although sparse, the current evidence suggests that MIF signaling may be beneficial in iatrogenic ICM but may promote alcoholic cardiomyopathy.

### 4.3 Autoimmune myocarditis

The autoimmune etiologies of inflammatory cardiomyopathies are well established. An early study found that Lewis rats with EAM exhibited elevated levels of MIF mRNA in the myocardium, which was reduced by treatment with human intact immunoglobulin (118). Additionally, it has been reported that treatment of rats with a neutralizing antibody to MIF inhibited onset and reduced severity of EAM (50). Using the same model in mice, we found that genetic ablation of MIF combined with dexamethasone treatment just prior to the acute phase of EAM reduced disease severity and prevented progression to DCM (unpublished data). The role of MIF in the development of ICM caused by autoimmune diseases has yet to be established.

### 4.4 Myocardial ischemia and infarction

While MI is not directly caused by immune cells, it is now understood that the immune response in the ischemic or infarcted heart plays a critical role in cardiac tissue damage, repair, and ultimately progression to HF. MIF is released by the myocardium and infiltrating leukocytes and impacts various aspects of myocardial ischemia and ischemia-reperfusion injury. MIF is reported to have both protective and proinflammatory effects in myocardial ischemia and infarction. Initially, cardiac cells release MIF in response to acute MI, and within one day post-MI, inflammatory cells become the primary source of MIF (147). Importantly, although the majority of studies reported that MIF has protective effects in MI, it has been postulated that the duration of ischemia influences the effects of MIF in MI pathogenesis (147).

More than two decades ago hypoxia and oxidative stress, two critical components in the pathogenesis of ischemia/reperfusion injury, were shown to induce secretion of MIF from cardiomyocytes (148, 149). Shortly thereafter, MIF overexpression was observed in cardiomyocytes and macrophages in a rat model of acute myocardial ischemic injury (150). Evidence suggests that MIF exhibits a cardioprotective role by activating AMPK signaling through the CD74/CD44 MIF receptor promoting energy conserving pathways, inhibiting apoptosis, suppressing inflammation, and reducing oxidative stress (15, 151–153). Accordingly, mouse hearts perfused with the MIF agonist MIF20 prior to no-flow ischemia and reperfusion showed improved post-ischemic left ventricular function, concomitant with increased cardiac MIF-AMPK activation and improved myocardial glucose uptake (151). The same study reported that MIF20 afforded protection in the hearts of mice subjected to left coronary artery occlusion and reperfusion, which significantly reduced infarcted myocardium (151). Additionally, stem cell-derived exosomes showed efficacy in treating injured cardiac tissue via aiding in repair and regeneration, and mesenchymal stem cell-derived exosomes engineered to contain MIF were reported to facilitate exosome-mediated cardioprotective effects in rats with acute MI (154, 155).

Most studies providing evidence for the protective role of MIF in MI limited ischemia to less than 30 minutes, but studies in which the duration of ischemia was increased to 60 minutes reported MIF to have a detrimental role in cardiac ischemia, suggesting time after injury as an important variable for MIF effects (106, 156–159). Of note, another recently published study reported that MIF was important for the protective effects of dexmedetomidine treatment in mice with ischemia-reperfusion injury, in which ischemia lasted 60 minutes (160). Moreover, studies attempting to dissect the opposing functions of MIF in MI reported that the protective effects were mediated via MIF and CXCR2 signaling in cardiac tissue-resident cells, which promoted survival and myocardial healing, whereas the detrimental effects of MIF were dependent on leukocyte-derived MIF and cardiac fibroblast-derived CCL2 to promote myocardial inflammatory infiltration (106, 156–159). Thus, it is conceivable that preventing leukocyte infiltration while maintaining cardiac-derived MIF secretion post-MI could prove beneficial.

Taken together, the evidence suggests that MIF agonism may be a promising therapeutic approach to improve patient outcomes, but conflicting reports warrant further investigation into the effect of MIF signaling at different stages of ischemia and MI.

### 4.5 Cardiac remodeling and heart failure

HF is an important complication of inflammatory cardiomyopathies and is associated with poor prognosis (3). The immune system plays a critical role in the progression to HF by influencing cardiac remodeling that leads to DCM, reduced ejection fraction, myocardial hypertrophy, and fibrosis (1). In cardiomyopathy patients, myocardial MIF expression was elevated in patients with ischemic cardiomyopathy compared with non-ischemic cardiomyopathy (161). Among patients with non-ischemic cardiomyopathy, myocardial MIF expression was elevated in those with chronic myocarditis and correlated with the degree of myocardial fibrosis (119). Additionally, MIF plasma levels associated with HF patient outcomes including pulmonary hypertension and death (120, 121). These data implicate MIF in HF pathogenesis and patient outcomes.

However, while clinical data implicated MIF as detrimental in HF caused by ischemia and inflammation, other studies suggested a protective function for MIF in cardiac remodeling in myocardial hypertrophy and fibrosis induced by pressure overload. Along these lines, in mice subjected to transverse aortic coarctation for 10 days, MIF-deficient mice showed significantly increased heart growth and cardiac fibrosis, suggesting that MIF reduced myocardial hypertrophy and fibrosis in pressure overload-induced cardiac hypertrophy (122). Similar findings were reported in mice subjected to abdominal aorta constriction-induced cardiac hypertrophy, where MIF deficiency worsened cardiac hypertrophy and decreased autophagy (123). More recently, MIF was reported to confer protection against pressure overload-induced cardiac hypertrophy and fibrosis by regulating the miR-29b-3p/HBP1 axis and the Smad3-miR-29b/miR-29c axis, respectively (162, 163). Moreover, soluble CD74 was demonstrated to inhibit MIF/CXCR4/AKT-mediated survival of myofibroblasts, suggesting that MIF prevents CD74-dependent necroptotic pathways (107).

Future research is needed to fully elucidate the mechanisms by which MIF influences cardiac remodeling and HF, and how different etiologies may influence the effect of MIF signaling in cardiac remodeling and HF.

### 4.6 Targeting MIF therapeutically

MIF has emerged as an attractive therapeutic target due to its role in several pathologies. One strategy is to use monoclonal antibodies targeting either MIF or MIF receptors (e.g., CD74). Early clinical studies using the anti-MIF monoclonal antibody imalumab reported a favorable safety profile in patients with advanced solid tumors (164). Similarly, nanobodies targeting MIF have been developed for sepsis treatment, some of which have shown promise (165). Small molecule inhibitors targeting MIF have been developed to prevent binding of MIF to CD74. The first small molecule inhibitor of MIF, ISO-1, provided proof of concept for significantly increasing the survival of mice with sepsis (166), reducing disease severity in mouse models of SLE (167), and delaying onset in a mouse model of autoimmune diabetes (168). Additionally, treatment with the MIF inhibitor ISO-1 reduced diabetic nephropathy in T2D and ameliorated pathological hallmarks, including hyperglycemia, increased inflammatory cytokines, and increased macrophage activation in the kidney (169). Originally developed as a phosphodiesterase inhibitor, the small molecule inhibitor ibudilast was found to allosterically inhibit MIF and showed efficacy in a phase II clinical trial of multiple sclerosis patients (170, 171). Glucocorticoids represent an indispensable option for treating inflammatory conditions, but dose-limiting adverse effects and glucocorticoid resistance limit their use. Considering the antagonistic function of MIF on the immunosuppressive function of glucocorticoids, inhibition of MIF could be a viable option for treating diseases commonly associated with glucocorticoid resistance.

## 5 Future directions

The role of MIF in inflammation, immune responses, and various disease pathologies such as autoimmune disorders and cancer has been increasingly recognized, yet several key areas remain underexplored. Future research on MIF offers the potential to unlock new therapeutic strategies and deepen our understanding of this critical cytokine (172). In the following we will discuss several promising directions where research into the role of MIF in ICM seems warranted.

### 5.1 Refining small molecule inhibitors of MIF

Despite the advancements made in understanding MIF’s role in disease, small molecule inhibitors of MIF remain a critical area of investigation. Molecules such as ISO-1 and 4-IPP already showed promise in preclinical models by inhibiting the tautomerase activity of MIF, which is thought to interfere with MIF’s pro inflammatory functions (173). Recent studies highlight that ISO-1, specifically targeting the tautomerase active site, effectively inhibits pro-inflammatory activities such as TNF release from macrophages, demonstrating significant protection in severe sepsis models (166)**​**. However, the challenge persists in improving the specificity and potency of these inhibitors while reducing off-target effects. Additionally, ongoing research highlights that targeting MIF through novel inhibitors not only affects the tautomerase active site but also opens possibilities for disrupting its interactions with key receptors such as CD74 and CXCR4. Studies showed that allosteric inhibitors, such as ibudilast, effectively alter MIF’s conformation, thereby inhibiting its enzymatic and biological activities while crossing the blood-brain barrier, a crucial factor for treating CNS related inflammatory conditions (174). Furthermore, dual inhibition strategies targeting both MIF and its homolog D-dopachrome tautomerase (MIF-2) are being explored for their potential synergistic effects in mitigating inflammatory and autoimmune responses.

### 5.2 MIF as a biomarker for inflammatory cardiomyopathy

One of the most promising avenues of MIF research is its potential use as a biomarker for various diseases. Elevated levels of MIF have been associated with the severity of inflammatory diseases and cancers, making it a potential prognostic tool. For instance, in autoimmune diseases such as SLE and RA, MIF levels correlate with disease severity, and in cancers, including colorectal cancer, high MIF levels are linked to poor patient outcomes (173, 175). Future research could focus on integrating MIF as a blood and cardiac tissue biomarker in clinical diagnostics, allowing for better risk stratification and monitoring of disease progression.

In the context of Chagas disease, MIF has demonstrated significant potential as a biomarker for progressive chronic cardiomyopathy. Elevated circulating levels of MIF in Trypanosoma cruzi-infected patients have been strongly associated with severe cardiac involvement, correlating with markers of myocardial damage such as left ventricular dysfunction and increased high-sensitivity C-reactive protein (59). Measuring MIF levels in blood offers an additional tool for identifying patients with severe heart pathology who may benefit from early intervention and specialized treatments. Such investigations could inform the development of novel therapeutic strategies aimed at reducing long-term morbidity and mortality in Chagas patients, which may be extended to other inflammatory cardiomyopathies.

### 5.3 MIF in inflammatory cardiomyopathy

MIF plays a critical role in immune regulation, and it is increasingly implicated in the pathogenesis of ICM. Cells of the innate and adaptive immune system play a significant role in the pathogenesis of ICM. MIF signaling in these cell types has been extensively studied. Still, the exact mechanisms by which MIF signaling influences adaptive and innate immune cell function in ICM is unclear. For example, CD4^+^ T cells are required for the development of myocarditis in CVB3-infected and EAM mice (85, 91, 92), and Th17 cells promote progression of ICM to DCM (86). MIF is an essential regulator of T cell activation, proliferation, and antigen-specific immune responses (96, 97, 176); yet the role of MIF in promoting these processes in ICM has still to be explored. Similarly, B cells are implicated in ICM (3, 101), and MIF influences B cell functions including chemotaxis and survival (102, 103). Nevertheless, the role of MIF in promoting pathogenic B cell responses in ICM remains unclear. Likewise, how MIF influences pathogenic or protective functions in other cell types involved in ICM remains to be elucidated. Future research aiming to uncover the function of MIF in cell types implicated in ICM is needed.

Emerging evidence highlights the role of genetic and epigenetic factors in modulating MIF expression and activity, potentially influencing susceptibility to inflammatory cardiomyopathies. As described earlier, the MIF -173G/C polymorphism, known to affect MIF gene expression, has been associated with various cardiovascular and inflammatory conditions, including RHD patients in some populations (143, 177). Although a direct link to ICM remains elusive, GWAS in RHD patients suggest that polymorphisms within immune related genes can predispose individuals to dysregulated immune responses, as seen in autoimmune heart diseases (178). These findings imply that MIF genetic variants could modulate the inflammatory milieu in cardiomyopathies, mirroring its role in other inflammatory conditions. Further exploration of these genetic variants, combined with epigenetic studies, could provide crucial insights into the mechanisms driving disease susceptibility and progression.

### 5.4 Experimental models of inflammatory cardiomyopathy

Experimental animal models have been instrumental in understanding the complex pathogenesis of ICM and testing novel therapeutic strategies. These models replicate key aspects of human myocarditis, including autoimmune and viral triggers and provide in-depth insights into disease mechanisms. Among the most widely used models is EAM. The EAM model is induced by immunizing susceptible rodent strains, such as BALB/c mice, with cardiac-specific antigens, for example myosin peptides emulsified in complete Freund’s adjuvant. This method triggers strong T cell-mediated autoimmune responses against cardiac tissue, effectively mimicking autoimmune myocarditis in humans. EAM can transition to DCM, making this model valuable for studying the chronic phases of ICM (179).

In virus-induced myocarditis, infection with CVB3 remains one of the most useful models. CVB3 infection induces acute myocarditis characterized by myocyte necrosis and inflammatory cell infiltration, which can progress to chronic myocarditis and DCM (179). Other viruses, such as adenoviruses and herpesviruses, have also been used to expand our understanding of viral-induced ICM. These models replicate the dual impact of direct viral cytotoxicity and immune-mediated injury, providing insights into the complex interplay of host and pathogen factors (179). Additionally, drug-induced cardiomyopathy models leverage agents such as doxorubicin to induce cardiac injury through oxidative stress and mitochondrial dysfunction. These models facilitate the investigation of iatrogenic ICM and the development of cardioprotective interventions (180).

Advancements in genetic and technological methodologies offer opportunities to refine these models. Transgenic and knockout rodents allow for the dissection of gene-specific contributions to ICM. The emergence of organ-on-a-chip technologies mimicking cardiac microenvironments represents a promising approach for high-throughput therapeutic testing and detailed mechanistic studies (181). These experimental models continue to be invaluable for advancing our understanding of ICM and these innovations aim to overcome the translational gap between animal models and human conditions. Integrating novel approaches such as transgenic models, diverse disease models, and organ-on-a-chip technologies will enhance their accuracy and translational relevance, paving the way for innovative therapeutic strategies and bridging the gap between preclinical research and clinical application.

## 6 Conclusions

MIF is a pleiotropic cytokine implicated in several human diseases. Experimental animal models and clinical data implicate MIF in the development and progression of ICM, cardiac remodeling, and heart failure. Gaps remain in our knowledge of ICM pathogenesis and diagnosis. Current treatment regimens for virus-negative ICM remain suboptimal, and no standard treatment for virus-positive ICM has been established. Evidence suggests that MIF inhibition could serve as a viable therapeutic strategy for certain inflammatory cardiomyopathies such as CCC, autoimmune myocarditis, and viral myocarditis. Therapeutic targeting of MIF is an active area of investigation for many human diseases. Importantly, timing and etiology appear to be critical in determining the function of MIF in ICM, and in general cardiac tissue injury associated with inflammation. Although still unclear, the current evidence suggests that some inflammatory cardiomyopathies (e.g., iatrogenic cardiomyopathy) may benefit from MIF signaling, which coincides with the reported function of MIF in studies investigating myocardial infarction and remodeling. Finally, future experimental and clinical studies will help to determine how MIF signaling affects cell-specific pathogenic functions in ICM (**Figure 1**), in addition to aiding in the development of novel therapeutics targeting MIF. Future research will aim to elucidate the mechanisms by which MIF affords risk or protection in ICM, as well as other heart conditions characterized by inflammation.
